# Facilitators and barriers to the transition from outpatient clinic visits to home-based check-ups for children being treated with growth hormone: a mixed-methods study

**DOI:** 10.1007/s00431-023-05408-z

**Published:** 2024-01-31

**Authors:** Anouk J. W. Remmits, Ghislaine A. P. G. van Mastrigt, Silvia M. A. A. Evers, Petra A. van Setten

**Affiliations:** 1 Amalia Children’s Hospital, Department of Paediatric Endocrinology, Radboud University Medical Centre, Nijmegen, The Netherlands; 2 CAPHRI, School for Public Health and Primary Care, Department of Health Services Research, Faculty of Health, Medicine and Life Sciences, Maastricht University, Maastricht, The Netherlands; 3 Trimbos Institute, Netherlands Institute of Mental Health and Addiction, Utrecht, The Netherlands

**Keywords:** Transition to homecare, Telemedicine, Paediatrics, Growth hormone treatment

## Abstract

Although the coronavirus disease 2019 (COVID-19) pandemic accelerated the adoption and expansion of telemedicine worldwide, little is known about the transition to home-based care for children. This study aims to investigate the facilitators and barriers to the transition from outpatient clinic visits to home-based check-ups (HBCU), for children being treated with growth hormone. A mixed-methods study was performed at Amalia Children’s Hospital (Radboud University Medical Centre, Nijmegen), consisting of questionnaires and semi-structured and focus group interviews. For the quantitative part, the Measurement Instrument for Determinants of Innovation (MIDI) was utilised to investigate the facilitators and barriers for the 81 participants regarding the transition to HBCU. The MIDI questionnaire is comprised of four domains: the innovation-, user-, organisation-, and the socio-political scale. Descriptive statistics were performed for analysing the questionnaires. For the qualitative part, interviews with 10 participants derived from the questionnaire and the two focus group interviews were conducted, to gain more in-depth information about the research topic, until data saturation was reached. The interviews were analysed by using the reflective thematic approach, starting with deductive coding and followed by inductive coding. Several facilitators were recognised in our study: procedural clarity, self-efficacy, convenience, patient-centred care, increased accuracy in height measurements, social support, client/patient satisfaction/cooperation, patient-centred care, the flexibility and adaptivity of HBCU, physical start-up period of HBCU, and a potential decrease in healthcare costs. However, several barriers were also noted in our study: poor compatibility with current practice, lack of consultation within the team, feeling of being less controlled by physicians, unsettledness of the organisation, an increased workload for the staff, and insufficient information communication technology (ICT) facilities.

*Conclusion*: This study revealed that HBCU have considerable benefits for both patients and healthcare professionals, from the standpoint of innovation, user, and socio-political points of view. The identified facilitators and barriers to HBCU should be taken into account when further steps of implementing HBCU are considered.

**Table:** 

**What is Known:** *• The Corona-Virus-Disease 2019 (COVID-19) pandemic has had an immense impact on health care worldwide. A substantial amount of the outpatient clinic visits for children treated with growth hormone was, as a result of the pandemic, transferred to online consultation. Transitioning paediatric growth hormone treatment to the home setting may be favorable for children and their parents/caregivers) as well for healthcare professionals.* *• Insights regarding facilitators and barriers is vital for the successful implementation and adoption of home-care technologies.*	
**What is New:** *• To our knowledge, we are first to report on and explicit the facilitators and barriers of the transition to home-based check-ups, via online consultation for children being treated with growth hormone.* *• Both children and healthcare professionals reported major facilitators and some minor barriers to the transition to home-based check-ups, illustrating their potential value. These facilitators and barriers should be considered while working towards implementation of home-based check-ups.*

**Supplementary Information:**

The online version contains supplementary material available at 10.1007/s00431-023-05408-z.

## Introduction

Since 2020, the coronavirus disease 2019 (COVID-19) pandemic has had an immense impact on healthcare, leading to a worldwide increase in the use of telemedicine for online consultation [1]. Telemedicine is the use of information communication technology (ICT) to deliver adequate remote healthcare and is a proper manner to deliver outpatient clinic care at a distance, particularly appropriate during a pandemic [2]. The Dutch State Institute for Public Health and Environment (RIVM) has noted a decline of 38.0% in hospital visits in 2020; approximately 12.0% of those postponed hospital appointments were transferred to online consultation [3]. Online consultation, via telemedicine, is an alternative, also in non-COVID-19 times, for paediatric endocrinologists to provide timely and efficient consultation [4, 5]. Furthermore, earlier studies showed that transitioning outpatient care to the home setting lowers the burden of disease for children [6]. We anticipate that the integration of telemedicine into paediatric clinical practice will increase, accelerated by the COVID-19 pandemic [7, 8]. Insight into the facilitators and barriers to the transition to home-based check-ups (HBCU) via video consultation is vital for its successful implementation and adoption. If the hindering factors are known, they can be corrected before the actual implementation and focus can be maintained on the facilitating factors, to encourage all stakeholders involved in the care process.

In the Netherlands, approximately 1/10,000 children annually start growth hormone (GH) treatment. This is in accordance with previous reports (1/4000–1/10,000) [9–11]. Several paediatric diseases, including but not limited to GH deficiency and resistance, result in short stature [12]. The primary goal of this treatment is to promote height velocity and to improve final height close to the patient’s target height. Reliable height and weight measurements are an essential part of outpatient clinic appointments because estimating the body surface area (BSA) is one of the cornerstones of GH dosing [13]. GH treatment requires adequate monitoring by a paediatric endocrinologist, including four appointments annually to adapt the GH dosage. In addition, attention is paid to general well-being and compliance, for which consultation at the outpatient clinic is not per se needed. These outpatient clinic visits are time-consuming for parents as well as for healthcare professionals. Whenever the height measurements of these children can be measured accurately in the home setting, at least part of the consultation can be transitioned to the home setting. In our recently published study, we found that parentally performed height measurements (in the hospital setting) strongly correlate to height measurements performed by outpatient clinic nurses, indicating that caregivers are able to accurately measure the height of their child at home [14]. Furthermore, earlier research in the field of obesity in children also revealed a strong correlation between parentally reported height measurements and measurements performed by observers [15].

Transitioning outpatient clinic visits for paediatric GH to HBCU is innovative and promising for future healthcare sustainability. Healthcare innovations may offer a solution for the increased workload in hospitals and cost-effectiveness when compared to physical appointments. In addition, transitioning outpatient clinic visits may have a positive impact on patients and families as it saves time, travelling, and money [16, 17]. This study aims to investigate the facilitators and barriers for relevant stakeholders towards transferring outpatient clinic visits (treatment as usual (TAU)) to HBCU in the future, for children treated with.

## Methods

### Study design

A mixed-methods study using a sequential design was performed at the Amalia Children’s Hospital, Radboud University Medical Centre (Radboudumc), a teaching hospital in Nijmegen, The Netherlands. The study consisted of a quantitative part (questionnaires) and a qualitative part (semi-structured and focus group interviews) and triangulation of data sources was used to get richer and fuller information about the research topic [18, 19]. The descriptive nature of this study facilitated its ability to identify barriers and facilitators [20]. Data was collected by the researcher AR (during the period of data collection a master’s student in Healthcare Policy, Innovation & Management at Maastricht University, and a first-year master’s student in Medicine at Radboud University, with interests in researching the transition to HBCU) and stored in Castor EDC. The researcher had no conflict of interest and had no prior treatment relationship with the participants. We followed The COnsolidated criteria for REporting Qualitative research (COREQ) and Good Reporting of A Mixed Methods Study (GRAMMS) checklists for reporting (Supplementary materials Tables 1 and 2) [21, 22].

### Participants

For the study, two groups of participants were composed. The first group (group A) consisted of healthcare professionals (HCPs)—physicians, outpatient clinic nurses (OCNs), GH instruction nurses, and the departmental management of the Amalia Children’s Hospital, Radboud University Medical Centre; and the second group (group B) consisted of children being treated with GH and their parents or caregivers. Inclusion criteria for both groups were being involved in GH treatment, and the ability to speak and read Dutch. The participants from group A were recruited in person from the outpatient clinic of the hospital or via email. In addition, two paediatric endocrinologists from a nearby hospital (Canisius Wilhemina Ziekenhuis), participated in this study. Participants from group B were all recruited in person during their outpatient clinic visits. Children under the age of 12 participated in the study together with their parents or caregivers, and children over 12 were allowed to choose whether they wanted to participate together with their parents or caregivers or alone (hereafter referred to as children & parents/caregivers). Informed consent was obtained once for the study, for group A either via email, oral agreement, or through a written informed consent form. In addition, written informed consent from the participants from group B was retrieved. Before the start of the study, all participants were asked whether they wanted to participate in the semi-structured interviews. Their decision was recorded through the informed consent form and questionnaire. Data collection took place in May and June 2021.

### Definition of HBCU

HBCU are comprised of several elements, to provide adequate home-based care for GH treatment. Children and their parents or caregivers receive instructions from an OCN about accurately measuring height and weight. The results of the height and weight measurements will be uploaded online before the online consultation and will be discussed with the physician during the online consultation. The content of the HBCU was similar to TAU, with the exception of physical examination including pubertal characteristics, blood tests, and skeletal age determination. Even while utilising HBCU, the child will continue to visit the physical hospital at least once a year, for blood tests, control of pubertal characteristics, and determination of the skeletal age. Checking these indicators via online consultation is impossible.

### Measurements

#### Quantitative part (questionnaires)

The evidence-based Measurement Instrument for Determinants of Innovations (MIDI) was used to investigate the facilitators and barriers to the transition of TAU to HBCU [23, 24]. The MIDI questionnaire is designed to improve the understanding of important aspects that may affect the implementation of healthcare innovations and consists of four domains: the innovation-, the user-, the organisation-, and the socio-political scale. Not all determinants of the questionnaire were suitable for the current phase of the HBCU; therefore, the researchers critically examined which questions were suitable for the current phase appropriate for the stakeholders (Supplementary materials Table 3).

The responses on the MIDI questionnaire were based on the Likert scale and ranged from totally disagree (1) to totally agree (5). Furthermore, two open questions were added to the questionnaire to gain more in-depth information about the personal (dis)advantages (determinant 8 of the MIDI questionnaire) to HBCU. No specific (dis)advantages were tested, to give the responders space to share their perspectives on these matters, and the following open-ended question was added: “If yes, which (dis)advantages?” [25]. The answers given in the open-ended questions were supplementary to the facilitators and barriers identified from the questionnaire of interviews. In addition, participants were asked to assess an overall grade (range 1–10) for HBCU. After the questionnaires were composed, they were discussed with a representative from the group, and minor adjustments were made. The participants had the option of completing the questionnaire on paper, online, or orally with the researcher.

#### Qualitative part (semi-structured and focus group interviews)

Semi-structured and focus group interviews were conducted to gain more in-depth information (supplementary to the questionnaires). For the semi-structured interviews, relevant stakeholders (from groups A and B) were identified via purposive sampling, and participants were included until data saturation was reached [26, 27]. Data saturation was attained when, in at least three consecutive interviews, no new information was revealed [28]. Topic lists were established before the interviews, based on the determinants of the MIDI questionnaire, to provide structure to the interviews. To represent the opinion of the children and their caregivers, the Children’s Advisory Board (CAB) and the Parents’ Advisory Board (PAB) of Amalia Children’s Hospital were included in the focus groups’ interviews. The CAB includes children undergoing treatment at Amalia Children’s Hospital and the PAB consists of parents of children being treated at the hospital. The participants from these groups are experienced in receiving hospital care and are trained to actively contribute to decisions within the hospital. The content of the interviews was tested with a representative of the focus groups. All interviews were conducted by the female researcher (AR). Field notes were taken by the researcher during the interviews.

### Data analysis

#### Quantitative part (questionnaires)

The Statistical Package for the Social Sciences (SPSS) 25.0 was utilised for the statistical analysis. Descriptive analyses (mean, standard deviation, and percentages) were computed to evaluate the facilitators and barriers to the transition from TAU to HBCU. We tested the internal consistency (Cronbach’s alpha) of our questionnaire to assess the reliability of the questionnaires [29]. Determinants for which ≥ 20% of the total number of responses answered “strongly disagree” or “disagree” were considered barriers and determinants for which ≥ 80% of the total number of responses answered “strongly agree” or “agree” were considered facilitators [30]. For this study, facilitators noted in both groups and barriers reported in one of the groups were discussed as facilitators and barriers to the transition from TAU to HBCU. The definitions of facilitators and barriers were based on the study of Bach-Mortensen and Verboom [31]. Factors were considered as facilitators if these promote the implementation or adoption of innovations and/or transitions in healthcare. Factors were considered as barriers if these factors impede the implementation of innovations and/or transitions in healthcare. The mean of the total grade (1–10) was calculated for the groups. Moreover, an inductive approach was used for analysing open-ended questions about personal advantages and disadvantages. Topics (personal advantages and disadvantages) noted at least two times by the children/parents or HCPs were determined as facilitators and barriers for HBCU.

#### Qualitative part (semi-structured interviews and focus group interviews)

The steps of the reflective thematic approach were used for the analysis [32, 33]. The first step of the reflexive thematic analysis was familiarisation with the data. The interviews were conducted by the female researcher (AR). In addition, field notes were taken by the researcher during the interviews to get familiarised with the data. The interviews were manually transcribed by the researcher (AR) in MS Word. After member-checked approval, the transcripts of the interviews were coded by the same researcher via Atlas.Ti.20. The researcher reviewed the acquired data critically, subsequently followed by the second step, coding of the data. A combined approach was used for the coding process [34]. The data derived from the interviews was supplementary to the data of the questionnaire and therefore was the MIDI questionnaire a starting point for the data analysis. Deductive codes were assigned based on the MIDI questionnaire [24]. In addition, inductive coding was applied to explore the data on important domains not identified in the deductive phase. The codes contained more than one word, so it is clearly understood what they mean as well. Each coded segment was iteratively read and coded. The third step of the reflexive thematic analysis was generating initial themes. This was done by critically assessing the retrieved codes, central concepts were sought under which the various codes could fall. This was a first step in identifying themes. The next step of the reflexive thematic analysis was reviewing and developing themes (fourth step). The themes were further adapted and developed by looking again at the coding and the data. This was also discussed with PvS. If necessary, certain themes that corresponded were merged, new themes were added, and duplications in the codes were removed. The fifth step of the reflexive thematic analysis was refining, defining, and naming the themes. The themes were divided into final themes and given descriptions and names. Based on this, a code tree was created (see Supplementary materials Fig. 1). The last (sixth) step of the reflexive thematic analysis was producing the report. Based on these themes (along with the previously collected data from the questionnaires), the data was represented in story form, and quotes that were supportive of the story were added to the text. The quotations derived from the analysis were translated directly from Dutch to English.

### Ethical considerations

Children and parents were informed about the study’s goals and procedures, after they were asked for written informed consent to collect and analyse data. The procedures followed were in accordance with the World Medical Association Declaration of Helsinki. The Medical Research Ethics Committee of Nijmegen determined that this study did not fall within the remit of the Dutch “Medical Research Involving Human Subjects Act” (No. 2021–7506). In addition, the study was approved by the FHML-*RE*C*-*commission (file no. FHML-REC/2021/026/HPIM.077) of Maastricht University.

## Results

### Baseline characteristics

**Fig. 1:**
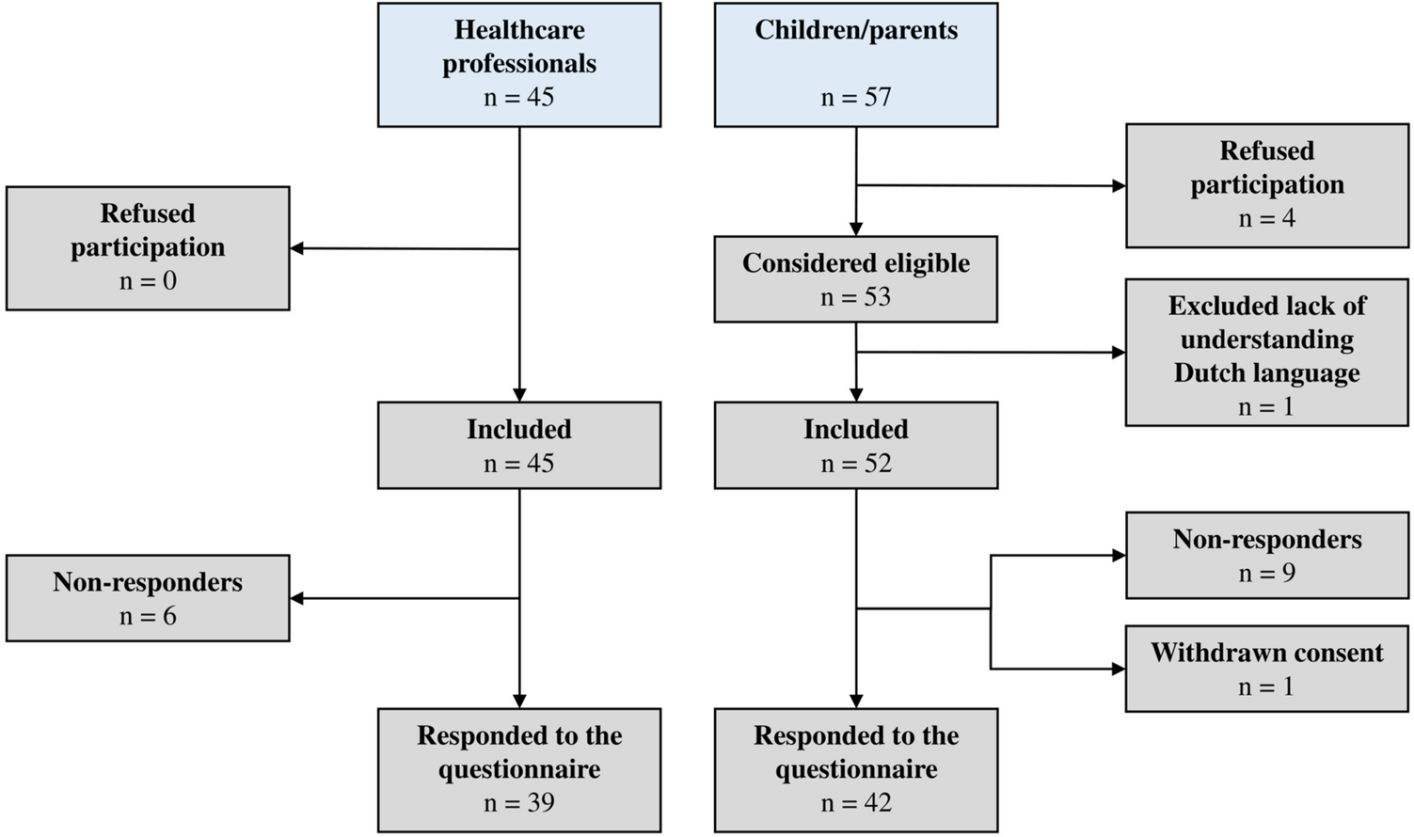
Flowchart of the participants in this study

In total, 102 participants were recruited for participation in this study, and 39 HCPs and 42 children/parents participated in the questionnaire (Fig. 1). Four children refused to participate in this study, one participant was excluded due to a language barrier and one participant withdrew consent because of negative feelings about HBCU in general. In total, 15 participants did not reply to the questionnaire resulting in a response rate of respectively 86.7% and 73.7% for group A (the HCPS) and group B (children/parents). The participants came from a wide range of various backgrounds (Tables 1 and 2). Ten semi-structured interviews were performed with participants from the questionnaire (with a response rate of 100% of the total number of participants approached for participating in the semi-structured interviews). In addition, two focus group interviews were conducted (Table 3). Data saturation was reached after nine interviews. Of the twelve interviews conducted, nine interviews were conducted face-to-face and three online. The interviews took place once and were audio-recorded, and their duration varied between 30 min and 1 h. After being transcribed, the summary of the transcript was sent to the interviewees/group for member-checking. Two interviewees provided feedback on the transcripts.

**Table 1:** Characteristics of the HCPs (group A) participating in the questionnaire

**Outcome (scale)**	**HCPs* n* (= 39)**
**Age (SD)**	41.49 (12.17)
**Gender, *n* (%)**	
** Female**	35 (89.7%)
** Male**	3 (7.7%)
**Discipline, *n (%)***	
** Physician**	11 (28.2%)
Paediatric endocrinologist*	6
** Paediatrician metabolic diseases**	1
** General paediatrician**	1
** Paediatric medical assistants**	3
** Outpatient clinic nurse**	19 (52.7%)
** Nurses**	6 (15.4%)
** Growth hormone injection instruction nurse**	5
** Nursing specialist (endocrine disorders)**	1
** Management of the outpatient clinic of Amalia Children’s Hospital**	3 (7.7%)
**Duration current job, *n (%)***	
** 0–1 year**	12 (30.8%)
** 1–3 years**	10 (25.6%)
** 3–5 years**	2 (5%)
** 5–10 years**	6 (17.9%)
** 10–20 years**	4 (10.3%)
** 20–30 years**	2 (5.1%)
** >30 years**	3 (7.7%)

Characteristics of the participants in the questionnaire

*HCPs* healthcare professionals, *SD* standard deviation

*Four paediatric endocrinologists were working at Amalia Children’s Hospital, two at the Canisius Wilhelmina Hospital Nijmegen

**Table 2:** Characteristics of the children (group B) participating in the questionnaire

**Outcome (scale)**	**Children *n* (= 42)**
**Age (SD)**	10.83 (3.35)
** <12 years**	23
** >12 years**	19
**Gender, *n* (%)**	
** Female**	24 (57.1%)
** Male**	18 (42.9%)
**Medical background*, n* (%)**	
** Syndromes associated with short stature**	12 (28.6%)
** Turner syndrome**	11
** Noonan syndrome**	1
** Skeletal dysplasia**	3 (7.1%)
** SHOX gene mutation**	2
** ACAN mutation**	1
** SGA**	12 (28.6%)
** SGA (without catch-up growth)**	9
** SGA (Silver-Russell** s**yndrome)**	3
** Endocrine disorders**	15 (35.7%)
** Isolated GH deficiency**	7
** GH resistance**	2
** Panhypopituitarism**	2
** GH deficiency with additional deficiencies (following oncological treatment)**	4
**Treatment duration, *n* (%)**	
** <1 year**	2 (4.8%)
** 1**–**2 years**	5 (11.9%)
** 2**–**3 years**	3 (7.9%)
** 3**–**5 years**	5 (11.9%)
** >5 years**	27 (64.3%)
**Travel distance to the hospital, *n* (%)**	
** <15 min**	4 (9.5%)
** 15**–**30 min**	3 (7.1%)
** 30**–**60 min**	22 (52.4%)
** 60**–**90 min**	9 (21.4%)
** >90 min**	4 (9.5%)

Characteristics of the participants in the questionnaire

*ACAN mutation* mutation in the aggrecan gene, *GH* growth hormone, *SD* standard deviation, *SGA* small for gestational age, *SHOX* short-stature homeobox

**Table 3:** Characteristics of the participants of the semi-structured and focus group interviews

**Speaker**	**(Sub)group**	**Gender**	**Age category**	**Work experience/treatment duration at Amalia Children’s Hospital**
**SP1**	Child (with 2 parents)	Female	<12 years	>5 years
**SP2**	Child (with 1 parent)	Female	<12 years	1–2 years
**SP3**	Outpatient clinic nurse	Female	40–50 years	2–3 years
**SP4**	Outpatient clinic nurse	Female	30–40 years	3–5 years
**SP5**	Growth hormone injection instruction nurse	Female	50–60 years	>30 years
**SP6**	Nursing specialist	Female	50–60 years	>30 years
**SP7**	Management of the outpatient clinic	Male	50–60 years	>30 years
**SP8**	Management of the outpatient clinic	Female	30–40 years	<1 year
**SP9**	Physician	Female	30–40 years	<1 year
**SP10**	Physician	Female	30–40 years	2–3 years
**FG1**	Parents Advisory Board (PAB)			
**FG2**	Children’s Advisory Board (CAB)			

Characteristics of the participants in the semi-structured and focus group interviews

*FG* focus group, *SP* speaker

The internal consistency of the questionnaires for the HCPs was questionable to good (intervention scale: *α* = 0.853; user scale: *α* = 0.790^1^; organisation scale: *α* = 0.692). Regarding the questionnaire for the children (and their parents/caregivers), the internal consistency was excellent for both the innovation scale (*α* = 0.973) and the user scale (*α* = 0.918). Both HCPs and children/parents reported more facilitators than barriers for HBCU (Tables 4 and 5) and several personal advantages and disadvantages were stated in the open-ended questions (Table 6). The coding tree of the interviews is shown in the supplementary materials (Supplementary materials Fig. 1). The intervention was highly rated (1–10) in both groups ((HCPs, 8.03 (95% CI 7.70–8.35); children/parents, 8.29 (95% CI 7.87–8.70)).

**Table 4:** Facilitators and barriers towards the transition TAU to HBCU for group A (HCPs)

	***HCPs (n* = *39)***		
**Determinant**	**Mean (SD)**	**TD/D**	**TA/A**
**Procedural clarity (I)** **Clarity of (the order of) the steps of HBCU that should be performed**	4.38 (1.02)	7.7%	**92**.**3%**
**Correctness (I)** **The degree to which HBCU are based on factually correct knowledge**	4.15 (1.09)	5.1%	74.4%
**Completeness (I)** **The degree to which the activities described in HBCU are complete**	3.92 (1.22)	**20.5%**	71.8%
**(Non-)complexity (I)** **The degree to which working with HBCU is complex**	4.36 (1.02)	7.7%	77.0%
**Compatibility (I)** **The degree to which HBCU are compatible with the values and working method in place**	3.64 (1.20)	**20**.**5%**	61.5%
**Observability (I)** **The visibility of the outcomes for the user is clear to the (end)user**	4.26 (0.91)	2.6**%**	74.3%
**Relevance for the patient (I)** **The degree to which the (end)user believes that the HBCU are relevant for themselves/or the children**	4.28 (1.00)	7.7%	76.9%
**Personal advantages/disadvantages (U)** **The degree to which using HBCU had advantages or disadvantages for the user themselves**			
** Personal advantages (facilitators)**	4.56 (0.68)	0	**89**.**7%**
** Personal disadvantages (barriers)**	2.33 (0.93)	**76**.**9%**	12.8%
**Outcome expectations (U)** **The perceived probability and importance of achieving the client objectives as intended by HBCU**			
** Feel less like a patient**^**a**^	4.08 (0.91)	2.6%	69.4%
** More control over the disease**	NA	NA	NA
** Fewer hospital visit**^**b**^	4.72 (0.57)	0	**94**.**4%**
** More efficient care**	4.54 (0.72)	2.6%	**92**.**3%**
** Potential decrease in healthcare costs**	4.31 (0.92)	7.7%	**84**.**6%**
** Be engaged in innovation**^**c**^	5.00 (0.00)	0	**100%**
**Professional obligation (U)** **The degree to which HBCU fit in with the tasks for with the (end)user feels responsible**	4.23 (0.99)	2.6%	74.3%
**Client/patient satisfaction (U)** **The degree to which the (end)user is satisfied with HBCU**			
** Patient**	4.51 (0.68)	0	**89**.**7%**
** Client**	4.26 (0.68)	0	**87**.**2%**
**Client/patient cooperation (U)** **The degree to which the (end)user wants to cooperate with HBCU**	4.38 (0.67)	0	**89**.**7%**
**Social support (U)** **The expected support from co-workers/friends/family with HBCU**	4.31 (0.89)	5.1%	**82**.**0%**
**Descriptive norm (U)** **The degree to which the (end)users use HBCU**	4.38 (0.82)	0	79.5%
**Subjective norm (motivation to comply)**^**d**^** (U)** **The influence of important others on the use of HBCU**	3.69 (1.24)	16.7%	55.6%
**Self-efficacy (U)** **The degree to which the (end)user can execute the activities from HBCU**	4.58 (0.60)	0	**87**.**2%**
**Knowledge (U)** **The degree to which the (end)user has the knowledge needed to use HBCU**	4.18 (0.91)	5.1%	76.9%
**Staff capacity (O)** **Adequate staffing in the department or in the organisation where HBCU will be used**	3.77 (1.22)	15.4%	59%
**Time available (O)** **The amount of time available to use HBCU as intended**	3.77 (1.09)	10.3%	56.4%
**Material resources and facilities (O)** **The presence of materials and other resources of facilities necessary for the use of HBCU as intended**	3.64 (1.09)	12.8%	51.3%
Unsettled organisation*(O) **The degree that other (organisational) changes are in progress, which potential can be obstacles to the process of implementing HBCU**	1.36 (0.49)	**64**.**1%**	35.9%

Facilitators and barriers per determinant for group A (HCPs) of the MIDI-questionnaires. The scores were based on the Likert Scale except for the unsettled organisation*. The facilitators (≥ 80% TA/A) and barriers (≥ 20% TD/D) are shown in boldface

*HBCU* home-based check-ups, *HCPs* healthcare professionals, *I* innovation scale, *MIDI* Measurement Instrument for Determinants of Innovations, *NA* not applicable, *O* organisation scale, *SD* standard deviation, *TA/A* totally agree/agree, *TD/D* totally disagree/disagree, *U* user-scale

*No (TA/A): 1, yes (TD/D): 2

^a^ = HCPs *n*: 36

^b^ = HCPs *n*: 3

**Table 5:** Facilitators and barriers towards the transition TAU to HBCU for group B (children/parents)

	***Children/parents (n* = *42)***		
**Determinant**	**Mean (SD)**	**TD/D**	**TA/A**
**Procedural clarity (I)** **Clarity of (the order of) the steps of HBCU that should be performed**	4.50 (1.09)	7.1%	**90**.**5%**
**Correctness (I)** **The degree to which HBCU are based on factually correct knowledge**	NA	NA	NA
**Completeness (I)** **The degree to which the activities described in HBCU are complete**	4.10 (1.27)	16.7%	73.8%
**(Non-)complexity (I)** **The degree to which working with HBCU is complex**	4.50 (1.02)	9.5%	**88**.**1%**
**Compatibility (I)** **The degree to which HBCU are compatible with the values and working method in place**	3.95 (1.08)	11.9%	71.4%
**Observability (I)** **The visibility of the outcomes for the user is clear to the (end)user**	4.17 (1.01)	2.4%	69%
**Relevance for the patient (I)** **The degree to which the (end)user believes that the HBCU are relevant for themselves/or the children**	4.55 (0.89)	4.8%	**90**.**5%**
**Personal advantages/disadvantages (U)** **The degree to which using HBCU had advantages or disadvantages for the user**			
** Personal advantages (facilitators)**	4.43 (0.97)	7.1%	**88**.**1%**
** Personal disadvantages (barriers)**	3.07 (1.39)	**52**.**4%**	35.7%
**Outcome expectations (U)** **The perceived probability and importance of achieving the client objectives as intended by HBCU**			
** Feel less like a patient**^**a**^	2.95 (1.25)	**28**.**6%**	31%
** More control over the disease**	3.02 (1.14)	**21**.**4%**	26.2%
** Fewer hospital visit**^**b**^	4.45 (0.92)	2.4%	**83**.**3%**
** More efficient care**	NA	NA	NA
** Potential decrease in healthcare costs**	NA	NA	NA
** Be engaged in innovation**^**c**^	NA	NA	NA
**Professional obligation (U)** **The degree to which HBCU fit in with the tasks for with the (end)user feels responsible**	4.33 (0.82)	4.8%	**88**.**1%**
**Client/patient satisfaction (U)** **The degree to which the (end)user is satisfied with HBCU**			
** Patient**	4.52 (0.77)	2.4%	**88**.**1%**
** Client**			
**Client/patient cooperation (U)** **The degree to which the (end)user wants to cooperate with HBCU**	4.62 (0.83)	2.4%	**90**.**5%**
**Social support (U)** **The expected support from co-workers/friends/family with HBCU**	4.60 (0.86)	7.1%	**88**.**1%**
**Descriptive norm (U)** **The degree to which the (end)users use HBCU**	4.00 (0.91)	4.8%	69.0%
**Subjective norm (motivation to comply)**^**d**^ **(U)** **The influence of important others on the use of HBCU**	4.50 (0.80)	2.4%	**85**.**7%**
**Self-efficacy (U)** **The degree to which the (end)user can execute the activities from HBCU**	4.81 (0.59)	2.4%	**95**.**2%**
**Knowledge (U)** **The degree to which the (end)user has the knowledge needed to use HBCU**	4.74 (0.73)	2.4%	**95**.**2%**
**Staff capacity (O)** **Adequate staffing in the department or in the organisation where HBCU will be used**	NA	NA	NA
**Time available (O)** **The amount of time available to use HBCU as intended**	NA	NA	NA
**Material resources and facilities (O)** **The presence of materials and other resources of facilities necessary for the use of HBCU as intended**	NA	NA	NA
Unsettled organisation*(O) **The degree that other (organisational) changes are in progress, which potential can be obstacles to the process of implementing HBCU**	NA	NA	NA

Facilitators and barriers per determinant for group B (children/parents) of the MIDI questionnaires. The scores were based on the Likert Scale except for the unsettled organisation*. The facilitators (≥ 80% TA/A) and barriers (≥ 20% TD/D) are shown in boldface

*HBCU* home-based check-ups, *HCPs* healthcare professionals, *I* innovation scale, *MIDI* Measurement Instrument for Determinants of Innovations, *NA* not applicable, *O* organisation-scale, *SD* standard deviation, *TA/A* totally agree/agree, *TD/D* totally disagree/disagree, *U* user-scale

*No (TA/A): 1, yes (TD/D): 2

^a^ = HCPs *n*: 36

^b^ = HCPs *n*: 3

**Table 6:** Responses to the personal advantages and disadvantages of the transition from TAU to HBCU

	**Personal advantages (facilitators)**	***n* (%)**	**Personal disadvantages (barriers)**	***n* (%)**
**HCPs (*n* = 39)**	Fewer physical appointments	31 (79.5%)	Less accurate measurements	12 (30.8%)
	Child-friendly check-ups in own environment	9 (23.1%)	No control of injection sites	8 (20.5%)
	Potentially decrease in healthcare costs	8 (20.5%)	Increased workload for staff	5 (12.8%)
	Increased efficiency in healthcare	5 (12.8%)	No personal contact with the patient	4 (10.3%)
	Easier planning for parents and child	5 (12.8%)	Too much work for the parents	4 (10.3%)
	Intensively involved in own care process	3 (7.7%)	Dependence on (ICT) technology	4 (10.3%)
	Suitable for children’s online world	2 (5.1%)	Not applicable for all patients	3 (7.7%)
			The shift from administrative tasks for staff	2 (5.1%)
			The difference in observation skills of HCPs	2 (5.1%)
**Children/parents (or caregivers) (*n* = 42)**	Fewer physical appointments	29 (69.0%)	Less physical contact	8 (19.0%)
	Effective and timesaving	6 (14.3%)	No other aspects (besides growth) checked	4 (9.5%)
	Less waiting time	4 (9.5%)	Live contact is easier	3 (7.1%)
	Reduced burden on the child	4 (9.5%)	Inaccuracy of measurements	3 (7.1%)
	Less physical control in pandemic times	3 (7.1%)	Treatment with other hormones not checked	2 (4.8%)
	Fewer absences from liabilities	3 (7.1%)	Less control by the caregivers	2 (4.8%)
	Accurate measurement & insight into data	3 (7.1%)	No ad hoc advice or actions	2 (4.8%)
	Appropriate to the target group	2 (4.8%)	Language barrier	2 (4.8%)

Responses to the personal advantages and disadvantages of the transition from TAU to HBCU. Items cited twice or more were considered as personal advantages (facilitators) and personal disadvantages (barriers)

*HBCU* home-based check-ups, *HCPs* healthcare professionals, *ICT* information communication technology, *TAU* treatment as usual

### Facilitators to the transition of TAU to HBCU (quantitative and qualitative part)

#### Innovation scale

The steps of HBCU were perceived as clear (procedural clarity) for both children/parents and HCPs and therefore the sole facilitating factor for both the HCPs and children/parents in the innovation scale.

#### User scale

Most of the facilitators for both HCPS and children/parents were recognised in the user scale. Starting with the personal advantages stated in the questionnaire: check-ups in one’s own environment, effective and plannable care, fewer absences from school and work, and reduced waiting and travel time. These advantages can be subsumed under an overarching term, namely convenience (for the patient and/or healthcare professionals). The following statements were given in the interview related to these personal advantages. Firstly, effective, and plannable care: “Because of HBCU, I don’t have to take time off work; I’m sometimes at peak times at work and it’s difficult to take time off then. In addition, my child does not have to stay home from school for long periods” [SP1]. Secondly, reduced waiting and travel time: “We come from far away and with these checks, it saves a lot of travel time” [SP2]. In addition, patient-centred care, tailored to the children’s needs, comprises several personal advantages. These personal advantages could be facilitators for the transition to HBCU, since children and parents/caregivers could be more willing to go through this transition more quickly by virtue of these personal advantages. One of these personal advantages is fewer physical appointments. The hospital is frequently perceived as an oppressive environment for children, which may cause anxiety. As stated in the interviews, reducing hospital visits may be beneficial for both parent and child and therefore can be a facilitating factor for the transition from TAU to HBCU. “We will certainly also have patients, those parents who simply find it very pleasant that they don't have to come to the hospital, children who are afraid of the hospital, for example who find it scary what happens there, and I can imagine that it is a lot less stressful for them to sit at home behind a computer, than having to go to the hospital every time” [SP10].

Moreover, the theme patient-centred care was further explored in the interviews and there were some important and potential facilitators recognised: the flexibility and adaptation of the HBCU to the stage of life, stage of treatment, and to the wishes and needs of the child. As stated from the interviews: “Do you have to make it completely concrete for each parent, the same for all parents, what should happen in the hospital and what should happen at home? Isn't it better to let it depend on how things go?” [FG1]. Therefore, HBCU should not be a fixed treatment option for all patients. Children with non-complex conditions may be more suitable for HBCU than children with complex conditions. “I think this lends itself perfectly to a customised approach because of course, you don’t have to plan this for years in advance. I'm not sure if it's going to work out, but let's give it a chance and that's fine, and if it turns out that it's not working out for one or both of the parties, then you can schedule the next follow-up appointment live. So, in that sense, you can be flexible” [SP9]. Moreover, a physical consultation at the start of treatment was considered vital. “It is important in the beginning to get to know the people, that they also get to know you and that you also know a little bit about what kind of people they are. That you also have some room for social talk, but also to build that bond.” [SP9]. Getting to know each other face to face is beneficial for the relationship between children and HCPs. “So, in a way, I am in favour of video calling, but as a third time or so. So, in the beginning, you get to know the doctor” [FG2]. Additionally, the HCPs argued that online consultation often gives them more information about the child and the interaction with caregivers is helpful. “Children are much more at ease in their environment. You get more out of children when you have a video consultation with them than when you see them here at the outpatient clinic in general. They are much more of an open book then” [SP6].

Another facilitator derived from the questionnaire is a tested outcome expectation, namely fewer hospital visits. This facilitator was further established in the interviews: “If we don't have to go to the hospital so often, that would be nice” [SP2].

Patient satisfaction and client/patient cooperation were both perceived as facilitators in the questionnaire. Furthermore, the social support during HBCU (assistance from friends, peers, or family) was perceived as sufficient in the questionnaire and therefore a facilitator for the transition from TAU to HBCU. In addition, it was stated in the questionnaire that both groups believe that they are capable of working (self-efficacy) with HBCU. In the interviews, it was argued that the current method of taking height measurements may be less accurate because of TAU. This since the measurements are performed by different OCNs, which may cause differences. “The impure, unreliable factor [of being constantly measured by someone else] is largely removed if you set this up properly at home” [SP6]. Performing those measurements at home, with sufficient and valid equipment, by one single observer (caregiver/parent) may potentially result in more reliable measurements and therefore may be a facilitator for the transition from TAU to HBCU.

#### Organisation scale

The organisation scale was requested only by the HCPs; however, the HCPs stated no facilitators in this category. In addition, one facilitator was reported in relation to the (socio-political) environment: a potential decrease in healthcare costs.

### Barriers and suggestions for tackling barriers to the transition from TAU to HBCU (quantitative and qualitative part)

#### Innovation scale

Perceived barriers in the innovation scale (from the questionnaire) for the HCPs were completeness and compatibility with current practice*.* It was argued in the interviews that despite the experiences gained with HBCU during COVID-19, HBCU still do not completely correspond to current practice compared with TAU, “The home measurements are now the thing we get stuck on when we want to do a remote consultation. Look, in the past year (2020 during COVID-19), we have of course kept all our appointments online, especially when we could not see patients. But after that, we did more remote consultations than before, also with the children receiving growth hormones. And I’ve always asked the parents if they can measure at home. But you can just see from the growth curve that it is not quite accurate yet” [SP10]. The participants stated in the questionnaire as a personal disadvantage that they are afraid that HBCU will be the start of a major change in their work and will potentially lead to an increase in workload. To tackle this barrier, it is mentioned in the interviews that it is important to show the HCPs the positive aspects of HBCU: “In health care, there will always be people who want to go along with new changes, and there will always be less enthusiastic people. It is also a question of doing it and gaining confidence in it” [SP6].

#### User scale

Several barriers were reported in the user scale; two of them were in the outcome expectations for the children/parents. These questions tested whether the children felt less like a patient and have more control over their disease due to HBCU, and a substantial number of the children disagreed on these topics. According to the criteria for the analysis, these factors may be perceived as barriers and were further requested in the interviews. There it was argued that the children (and their parents/caregivers) did not feel like a patient because they did not feel sick during their GH treatment. This indicates the partially non-complexity of the children treated with GH and could be therefore a facilitating factor for the transition of TAU to HBCU: “My child is not ill; we are treated with growth hormone to grow taller but do not experience it as being ill” [SP1]. As stated in the personal disadvantages as a barrier was, no control of other medical problems was often mentioned by the children with complex conditions (for example oncological treatment or panhypopituitarism). This may indicate that there is a difference between the children with complex conditions and those with non-complex conditions regarding the applicability of HBCU and the tested outcome expectations.

Furthermore, several disadvantages (barriers) were based on the feeling of having less control. HCPs and children/parents are afraid that in HBCU important aspects will be missed by physicians, and that there is no control of the growth injection sites. “In general, I am a bit worried about issues overlooked if you visit a doctor less often” [FG1]. The HCPs also reported less accurate measurements as a disadvantage of HBCU. However, this was also stated as a facilitator so therefore it was further explored during the interviews. It was argued by several participants that performing home measurements is a matter of proper training. “You can check (for example by having the measurements taken at home and then at the outpatient clinic) whether the measurements are done properly. If this is the case, you can still instruct the parents, because they are still in the learning process. You can then ask the parents to demonstrate the measurement and give them tips” [SP9].

“Some parents can be insecure: you can empower them in the learning process by saying, “Oh, there’s not much difference. The learning-working path is important; parents gain confidence that they can do it and we gain confidence in parents, and then it is very easy to do, I think” [SP9]. The HCPs reported another barrier, the lack of consultation within the team. HCPs also stated the importance of the opinions of their peers about HBCU. The feelings of the team are of paramount importance to these participants.

#### Organisation scale

The unsettledness of the organisation was classified as a barrier in the questionnaire and therefore further defined in the interviews. It was confirmed that there are several ongoing changes in the hospital: “A lot is constantly changing in the outpatient department and the administrative pressure has increased over the years. A lot is being launched and initiated at the outpatient clinic, but will this also work, and will there be an interim evaluation? It is important to get everyone on board so that it can work properly” [SP3]. However, some participants argued that HBCU contribute to these changes, as they correspond to the implementation of HBCU: “There are many changes in the hospital, but these are precisely the changes that encourage this [innovation]” [SP6]. “This innovation fits nicely into the transition we are going through as an outpatient clinic” [SP7].

In addition, an increase workload for the staff was another reported barrier (as personal disadvantage) related to the organisation scale, which might hinder HBCU. This was also stated in the interviews: “Sometimes you notice, [at the department], that there is not that much time, especially when there is a lot of absence due to illness, and then it is a bit more difficult. But I do think that if there is space and you plan well t, it can be used, and it should be feasible” [SP4]. The capacity was not a barrier, however, the planning of available staff available must be sufficient: “There will always be peaks when you are short of manpower, but you must plan and combine this efficiently” [SP4]. Another barrier, stated as personal disadvantage, related to the organisation scale was an insufficient ICT network. HBCU are highly dependent on the ICT network and must therefore work properly for HBCU to be successful. “Good ICT-facilities (internet connection, etc.) are needed for online consultations” [SP10].

## Discussion

This is the first mixed-methods study to examine the facilitators and barriers to the transition from TAU to HBCU for children being treated with GH. Both HCPs and children (including parents/caregivers) reported predominantly facilitators. Many of the facilitators were mentioned in the user scale: self-efficacy, convenience, potentially increased accuracy in height measurements, social support, client/patient satisfaction and cooperation, patient-centred care, the flexibility of HBCU, and a physical start-up period. In addition, non-complex cases were perceived as being more suitable for HBCU than complex cases and therefore HBCU must be tailored to the child’s needs. The remaining facilitators (in the other scales) were related to the procedure and a potential decrease in healthcare costs. Although several barriers were recognised: compatibility with current practice, increased workload for the staff, insufficient ICT network, and the unsettledness of the organisation. In addition, two tested outcome expectations may be perceived as barriers; however, in the context of this study, these factors may even be facilitators. The non-complexity of the condition of many of the children ensures that HBCU suit the (in general non-complex) target group. Overall, this study showed that all stakeholders were highly positive about HBCU, and several relevant comments were made in relation to the implementation of HBCU.

The reported facilitators and barriers are in line with existing evidence regarding the facilitators and barriers to the transition from TAU to HBCU. The study of van Wijngaart et al. [35] notes several facilitators and barriers to the implementation of eHealth innovations in daily practice. The convenience, compatibility, physicians feeling less in control, and a malfunctioning ICT network are the main corresponding facilitators and barriers between our study and the study of van Wijngaart et al. [35], although the political context was requested more predominantly in this study and therefore divergent from our study. Furthermore, the convenience of HBCU (reduced waiting time and being user-friendly), along with its flexibility and being patient-centred, is reported facilitators in other studies and is in accord with our findings [36–38]. In addition, earlier studies have suggested that parents can perform measurements and online consultations at home [36, 39]. These factors align with the facilitators of self-efficacy and the possibility of performing accurate measurements at home recognised in our study. Insufficient planning, the possibility of missing medical aspects (concerns about lack of physical examination), and inadequate ICT facilities were the perceived barriers of HBCU which are in accordance with previous research [40]. The unsettledness of the organisation was reported in other studies to be a barrier to the implementation of innovations (as HBCU); however, as suggested, the unsettledness may be in line with HBCU [41].

Insight into the facilitators and barriers is of fundamental basis for implementing healthcare innovations (as HBCU). If these factors, especially barriers, are considered before the implementation and adoption, they may positively influence the outcome of the implementation. Flexibility and adaptation to the patient’s stage of life, treatment, and their needs and wishes are crucial factors in determining whether to implement the transition from TAU to HBCU. Pubertal characteristics for example cannot be investigated via online consultation. In addition, children with more complex issues (for example children being treated with GH because of panhypopituitarism) may not be the most suitable target group for HBCU. Accordingly, HBCU should not become the standard for all children being treated with GH. However, HBCU may work perfectly for children being treated primarily with GH (for example children with an isolated GH deficiency or SGA). Regarding the barriers, investing in good ICT facilities and efficient staff planning will advance the implementation of HBCU. Furthermore, it is important to take into consideration that HBCU differ substantially from TAU during the implementation and transition.

Our study has five strengths. First, data triangulation was used, via multiple (both quantitative and qualitative) sources, which improved the credibility of our findings [42]. Second, a prospective research design was chosen to investigate the research topic, which provided more information about our research topic [43]. Especially since COVID-19 played a pivotal role in the implementation and adoption of telemedicine innovations in healthcare. Third, a validated questionnaire was the foundation of the study. The MIDI questionnaire is widely investigated and used for identifying factors that may affect healthcare innovations and its implementation and therefore this questionnaire was chosen as the foundation of our study [24]. Fourth, the diversity of the different stakeholders participating in the study represented the various groups involved towards the transition to TAU and resulted in a multidisciplinary approach of the research topic. Fifth, the participants in this study covered a wide range of diverse backgrounds. Especially, the various medical backgrounds of the children participating demonstrated the difference between the non-complex and complex conditions of GH-treated children. As such, the non-complex group may be more suitable for HBCU. Despite these various strengths, five limitations were recognised in our study. First, several determinants of the questionnaire approached the cut-off values set for facilitators and barriers. These items did not reach the classification threshold for barriers or facilitators, and this may influence the implementation and adoption of HBCU. However, the qualitative approach of our study addresses this limitation. Second, presumably because several items of the questionnaire were deleted, the reliability score for one group was lower. Third, approximately 10% of the total population of children treated with GH at Radboud University Medical Centre participated in this study; this could be a potential threat to the generalisability of our study. We tried to circumvent this by including GH-treated children with various causative medical conditions. Fourth, purposive sampling was used to select participants for the semi-structured interviews, which may have resulted in a selection bias. However, we assume that the likelihood of selection bias is limited because the data gathered in the interviews was in line with the data from the questionnaires. Fifth, we did not ask our participants about their previous experiences with telemedicine. This could be a potential threat to the validity of our study.

Insight into the facilitators and barriers to the successful implementation and adoption of HBCU is crucial. HBCU might yield considerable benefits for both patients and HCPs in terms of convenience, delivering care tailored to patient needs, possibly more accurate height measurements, providing even more information to HCPs in comparison with TAU, and cost savings. HBCU should be flexible to the different stages of treatment, stages of life, and the wishes and needs of both children and parents. Whenever height and weight measurements at home prove reliable, we do think that our findings are transferable to other paediatric patient groups, at least partially (for example children with celiac disease, metabolic diseases). Notwithstanding the benefits of HBCU, several barriers are noted, which should be considered and actively monitored while implementing HBCU. It might be valuable for further research to investigate the first experiences with HBCU and to fine-tune the process during the implementation phase of HBCU. In conclusion, this study revealed the potential of HBCU for future healthcare.

## Supplementary Information

Below is the link to the electronic supplementary material.

Supplementary file1 (DOCX 66 KB)

## Data Availability

The raw quantitative data that support the findings of this study are available on request from the corresponding author (Petra van Setten). The data are not publicly available due to restrictions(they contain information that could compromise the privacy of research participants).

## Footnotes

1 One item (determinant 9; be engaged in innovation) was deleted for calculating the internal consistency because the group to which this question was put was too small to be included in the internal consistency analysis.
