# Correction to: Facilitators and barriers to the transition from outpatient clinic visits to home-based check-ups for children being treated with growth hormone: a mixed-methods study

**DOI:** 10.1007/s00431-025-06036-5

**Published:** 2025-03-01

**Authors:** Anouk J. W. Remmits, Ghislaine A. P. G. van Mastrigt, Silvia M. A. A. Evers, Petra A. van Setten

**Affiliations:** 1 Amalia Children’s Hospital, Department of Paediatric Endocrinology, Radboud University Medical Centre, Nijmegen, The Netherlands; 2 CAPHRI, School for Public Health and Primary Care, Department of Health Services Research, Faculty of Health, Medicine and Life Sciences, Maastricht University, Maastricht, The Netherlands; 3 Trimbos Institute, Netherlands Institute of Mental Health and Addiction, Utrecht, The Netherlands

**Correction to: European Journal of Pediatrics (2024) 183:1857–1870.**

10.1007/s00431-023-05408-z

In the original article, three references of the method section were accidentally not inserted.

The following references are added to the methods section (Qualitative part (semi-structured interviews and focus group interviews):

32. Braun, V., & Clarke, V. (2019). Reflecting on reflexive thematic analysis. Qualitative Research in Sport, Exercise and Health, 11(4), 589–597. 10.1080/2159676X.2019.1628806

33. Braun, V., & Clarke, V. (2006). Using thematic analysis in psychology. Qualitative Research in Psychology, 3(2), 77–101. 10.1191/1478088706qp063oa

34. Proudfoot, K. (2023). Inductive/Deductive Hybrid Thematic Analysis in Mixed Methods Research. Journal of Mixed Methods Research, 17(3), 308–326. 10.1177/15586898221126816

The original article has been corrected.

