# Supplementary material for: Facilitators and barriers to the transition from outpatient clinic visits to home-based check-ups for children being treated with growth hormone: a mixed-methods study
Source: Eur J Pediatr. 2024 Jan 31;183(4):1857–70. doi: 10.1007/s00431-023-05408-z (PMC11001668; doi:10.1007/s00431-023-05408-z)
Supplement: Supplementary file 1 — Supplementary file1 (DOCX 66 KB) [file 431_2023_5408_MOESM1_ESM.docx]

**Supplementary materials**

**Table of contents**

[Supplementary materials Table 1: Consolidated criteria for reporting qualitative research (COREQ): a 32-item checklist for interviews and focus groups 1](#_Toc155693763)

[Supplementary materials Table 2: Checklist of items for the Good Reporting of A Mixed Methods Study (GRAMMS) guideline 3](#_Toc155693764)

[Supplementary materials Table 3: Overview of determinants of the questionnaires and interviews 4](#_Toc155693765)

[Supplementary materials Figure 1: Code tree of the semi-structured and focus group interviews 6](#_Toc155693766)

## Supplementary materials Table 1: Consolidated criteria for reporting qualitative research (COREQ): a 32-item checklist for interviews and focus groups

| **Item No. and Topic** | **Guide Questions/Description** | **Reported on Page No.** |
| --- | --- | --- |
| Domain 1: Research team and reflexivity | | |
| Personal characteristics | | |
| 1. Interviewer/facilitator | Which author/s conducted the interview or focus group? | 5 |
| 2. Credentials | What were the researcher’s credentials? | 5 |
| 3. Occupation | What was their occupation at the time of the study? | 5 |
| 4. Gender | Was the researcher male or female? | 7 |
| 5. Experience and training | What experience or training did the researcher have? | NA |
| Relationship with participants | | |
| 6. Relationship established | Was a relationship established prior to study commencement? | 5 |
| 7. Participant knowledge of the interviewer | What did the participants know about the researcher? | NA |
| 8. Interviewer characteristics | What characteristics were reported about the interviewer/facilitator? | 5 |
| Domain 2: Study design | | |
| Theoretical framework | | |
| 9. Methodological orientation and Theory | What methodological orientation was stated to underpin the study? | 5 |
| Participant selection | | |
| 10. Sampling | How were participants selected? | 5 |
| 11. Method of approach | How were participants approached? | 5 |
| 12. Sample size | How many participants were in the study? | 9 |
| 13. Non-participation | How many people refused to participate or dropped out? Reasons? | 9 |
| Setting | | |
| 14. Setting of data collection | Where was the data collected? | 5 |
| 15. Presence of non-  participants | Was anyone else present besides the participants and researchers? | NA |
| 16. Description of sample | What are the important characteristics of the sample? | 9-11 |
| Data collection | | |
| 17. Interview guide | Were questions, prompts, guides provided by the authors? Was it pilot tested? | 7 |
| 18. Repeat interviews | Were repeat interviews carried out? If yes, how many? | 9 |
| 19. Audio/visual recording | Did the research use audio or visual recording to collect the data? | 9 |
| 20. Field notes | Were ﬁeld notes made during and/or after the interview or focus group? | 7 |
| 21. Duration | What was the duration of the interviews or focus group? | 9 |
| 22. Data saturation | Was data saturation discussed? | 7 |
| 23. Transcripts returned | Were transcripts returned to participants for comment and/or correction? | 9 |
| Domain 3: analysis and ﬁndings | | |
| Data analysis | | |
| 24. Number of data coders | How many data coders coded the data? | 8 |
| 25. Description of the coding tree | Did authors provide a description of the coding tree? | Supplementary materials |
| 26. Derivation of themes | Were themes identiﬁed in advance or derived from the data? | 8 |
| 27. Software | What software, if applicable, was used to manage the data? | 8 |
| 28. Participant checking | Did participants provide feedback on the ﬁndings? | NA |
| Reporting | | |
| 29. Quotations presented | Were participant quotations presented to illustrate the themes/ﬁndings? Was each quotation identiﬁed? | 14-18 |
| 30. Data and ﬁndings consistent | Was there consistency between the data presented and the ﬁndings? | 14-18 |
| 31. Clarity of major themes | Were major themes clearly presented in the ﬁndings? | NA |
| 32. Clarity of minor themes | Is there a description of diverse cases or discussion of minor themes? | NA |

## Supplementary materials Table 2: Checklist of items for the Good Reporting of A Mixed Methods Study (GRAMMS) guideline

| **Mixed methods reporting** | |
| --- | --- |
| **GRAMMS guideline** | **Location in manuscript where items are reported** |
| 1) Describes the justification for using a mixed methods approach to the research question | Methods (p. 5-8) |
| 2) Describes the design in terms of the purpose, priority and sequence of methods | Methods (p. 5-8) |
| 3) Describes each method in terms of sampling, data collection and analysis | Methods (p. 5-8) |
| 4) Describes the integration of the quantitative and qualitative components | Methods (p. 5-8) |
| 5) Describes any limitation of one method associated with the presence of the other method | Discussion (p. 19-21) |
| 6) Describes any insights gained from mixing or integrating methods | Results (p. 9-18) |

## Supplementary materials Table 3: Overview of determinants of the questionnaires and interviews

| Determinant | HCPs | Children/parents (or caregivers) | Children’s – and Parents’ Advisory Boards of Amalia Children’s Hospital |
| --- | --- | --- | --- |
| *Procedural clarity* | ✓ | ✓ | ✓ |
| *Correctness* | ✓ | 🗶 | 🗶 |
| *Completeness* | ✓ | ✓ | ✓ |
| *(Non-) complexity* | ✓ | ✓ | ✓ |
| *Compatibility* | ✓ | ✓ | ✓ |
| *Observability* | ✓ | ✓ | ✓ |
| *Relevance for the patient* | ✓ | ✓ | ✓ |
| *Personal benefits/drawbacks* | ✓ | ✓ | ✓ |
| *Outcome expectations* | ✓ | ✓ | ✓ |
| *Professional obligation* | ✓ | ✓ | ✓ |
| *Client/patient satisfaction* | ✓ | ✓ | ✓ |
| *Client/patient cooperation* | ✓ | ✓ | ✓ |
| *Social support* | ✓ | ✓ | ✓ |
| *Descriptive norm* | ✓ | ✓ | ✓ |
| *Subjective norm (motivation to comply)* | ✓ | ✓ | ✓ |
| *Self-efficacy* | ✓ | ✓ | ✓ |
| *Knowledge* | ✓ | ✓ | ✓ |
| *Awareness of content of innovation* | 🗶 | 🗶 | 🗶 |
| *Formal ratification by management* | 🗶 | 🗶 | 🗶 |
| *Replacement when staff leave* | 🗶 | 🗶 | 🗶 |
| *Staff capacity* | ✓ | 🗶 | 🗶 |
| *Financial resources* | 🗶 | 🗶 | 🗶 |
| *Time available* | ✓ | 🗶 | 🗶 |
| *Material resources and facilities* | ✓ | 🗶 | 🗶 |
| *Coordinator* | 🗶 | 🗶 | 🗶 |
| *Unsettled organisation* | ✓ | 🗶 | 🗶 |
| *Information accessible about use of the innovation* | 🗶 | 🗶 | 🗶 |
| *Performance feedback* | 🗶 | 🗶 | 🗶 |
| *Legislation and regulations* | 🗶 | 🗶 | 🗶 |

*Overview of the determinants of the MIDI-questionnaire that were included for the questionnaire and the semi-structured interviews for both HCPs and children/parents (or caregivers). HCPs=healthcare professionals.* *MIDI=Measurement Instrument for Determinants of Innovations.*

## Supplementary materials Figure 1: Code tree of the semi-structured and focus group interviews

*HBCU=home-based check-ups. ICT=information communication technology. TAU=Treatment as usual.*
